# Low-Cost Modification to Enhance Bone Marrow Aspiration Efficiency: A Technical Report

**DOI:** 10.7759/cureus.76612

**Published:** 2024-12-30

**Authors:** Siddharth Jain, Hardik Kharbanda, Priya Maheshwari, Aasim Khan, Sonal Mishra

**Affiliations:** 1 Orthopedics, All India Institute of Medical Sciences, Bhopal, Bhopal, IND; 2 Anesthesiology, All India Institute of Medical Sciences, Bhopal, Bhopal, IND

**Keywords:** bone marrow aspiration, high-volume aspirate collection, innovative modification, iv drip set, low-cost apparatus

## Abstract

Bone marrow aspiration is an essential procedure for diagnosing hematological conditions and therapeutic applications, such as bone marrow aspirate concentrate (BMAC) therapy for non-union of bones. This report presents a case of a 35-year-old male with femoral non-union treated with BMAC, requiring the collection of a large bone marrow aspirate volume. A novel, low-cost modification to the standard bone marrow aspiration apparatus was developed using components from an intravenous (IV) drip set, including a rubber bulb connector and a luer lock connector. This modification enhanced suction efficiency and facilitated the safe collection of 90 ml of bone marrow aspirate in a single session. The procedure was completed without complications, and the cost of modification was minimal. This case demonstrates the potential of simple, low-cost innovations to improve procedural efficiency, particularly in resource-limited settings.

## Introduction

Bone marrow aspiration is a minimally invasive intervention to evaluate various hematological abnormalities. Lately, bone marrow aspirate concentrate (BMAC) has been used to address non-union and delayed union [1].

Moyal et al., in their systematic review, have depicted that most of the studies required 60 to 180 ml of bone marrow aspirate to address the non-union of long bones [2].

Obtaining such a high amount of bone marrow aspirate sometimes becomes tedious. We have devised an innovative, low-cost modification for obtaining bone marrow aspirate to solve this issue.

## Technical report

A 35-year-old male patient presented with non-union of the femur 18 months post-fracture. The treating team planned to use BMAC to promote bone healing. A large volume of bone marrow aspirate was required, prompting the use of a modified aspiration apparatus. 

The standard steps of collecting bone marrow aspirate are followed [3].

However, the apparatus has been modified and can be prepared following the steps below.

**Figure 1:**
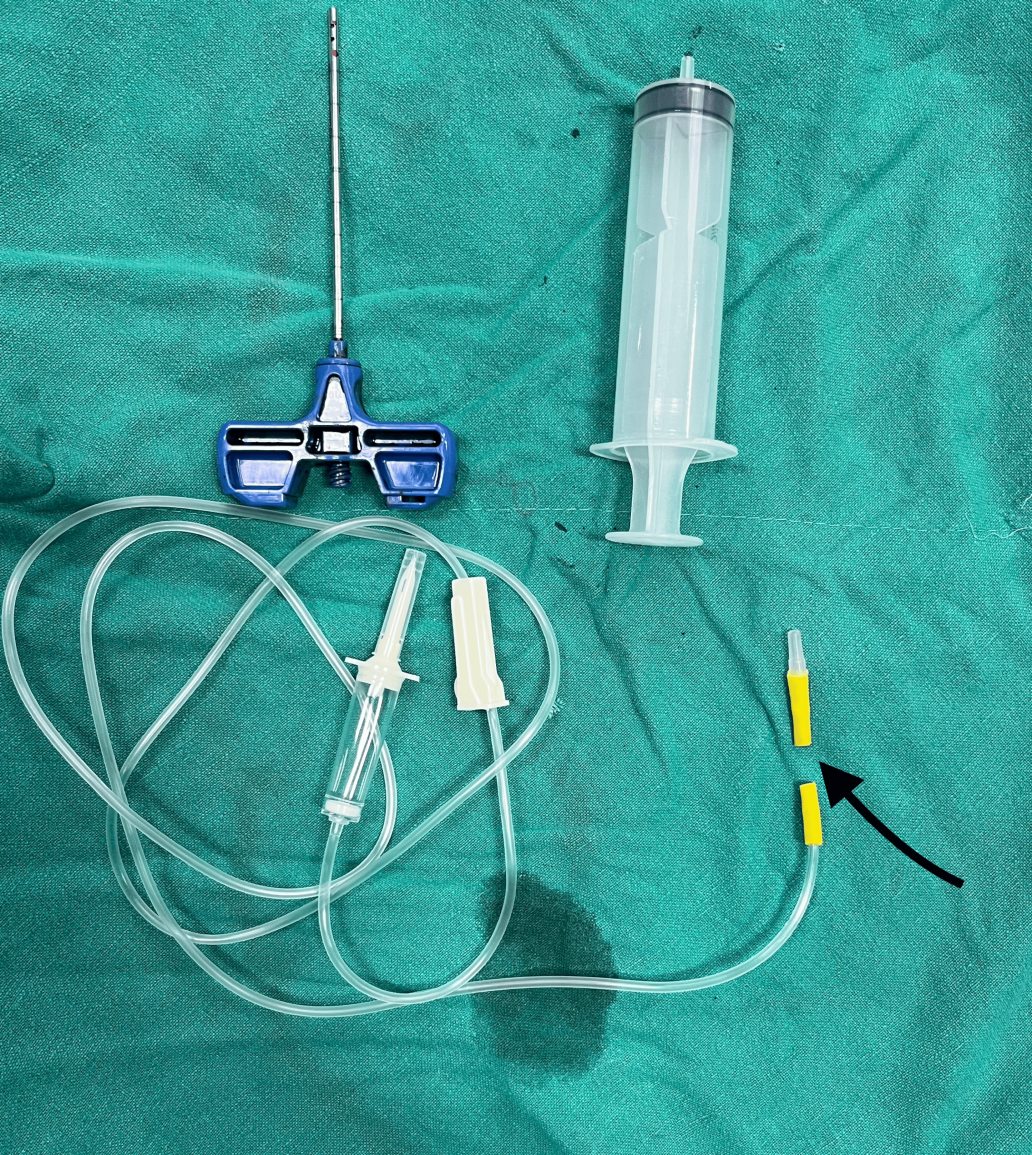
IV drip set is cut at its rubber bulb connector part (indicated by the black arrow) IV: intravenous

The intravenous (IV) drip set is cut at its rubber bulb connector part, as shown in Figure 1.

**Figure 2:**
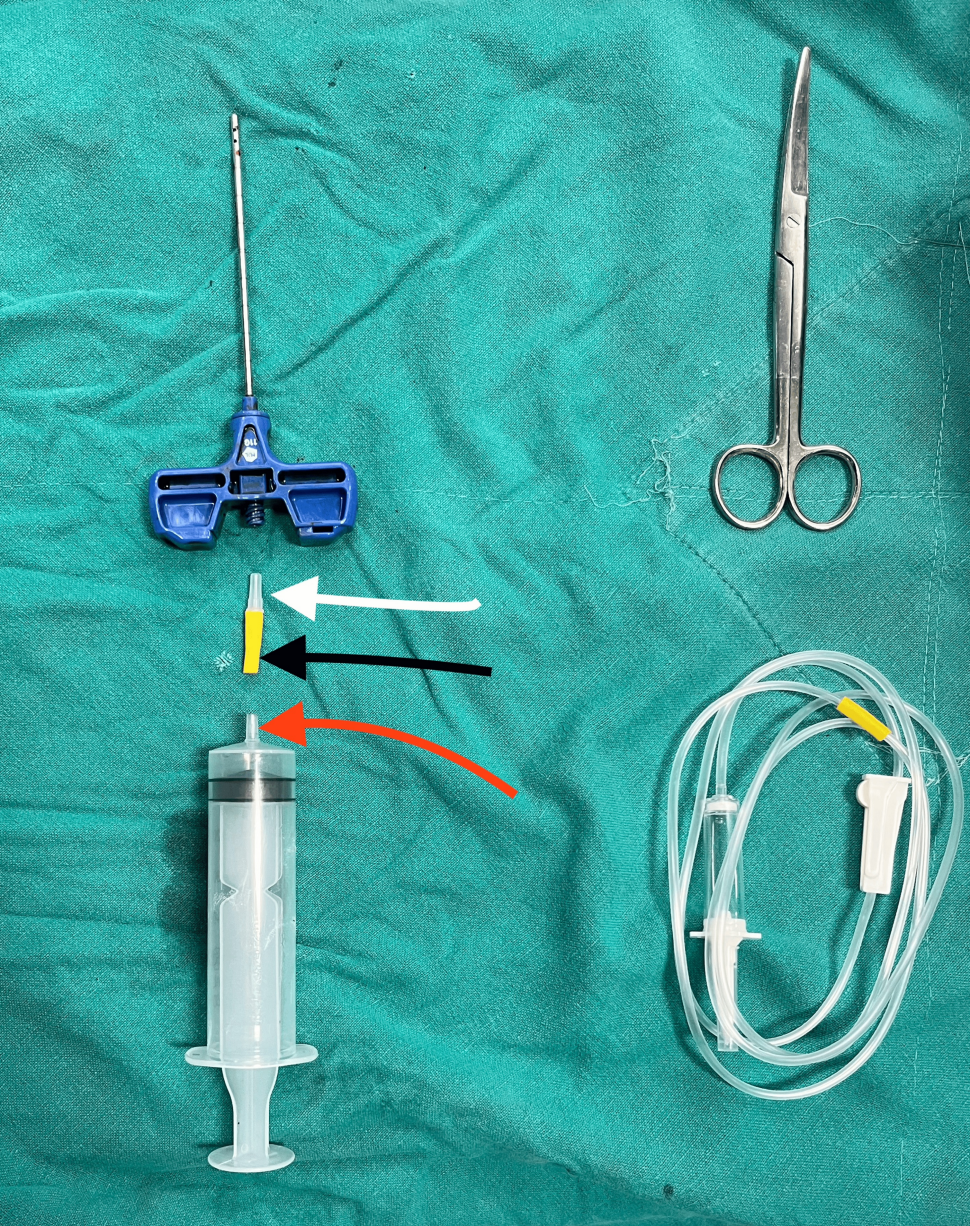
Parts of the assembly The red arrow denotes the syringe's hub; the black arrow indicates the rubber bulb part of the IV set; and the white arrow represents the luer connector part. IV: intravenous

Figure 2 depicts parts of the assembly. The red arrow denotes the syringe's hub; the black arrow indicates the rubber bulb part of the IV set; and the white arrow represents the luer connector part.

**Figure 3:**
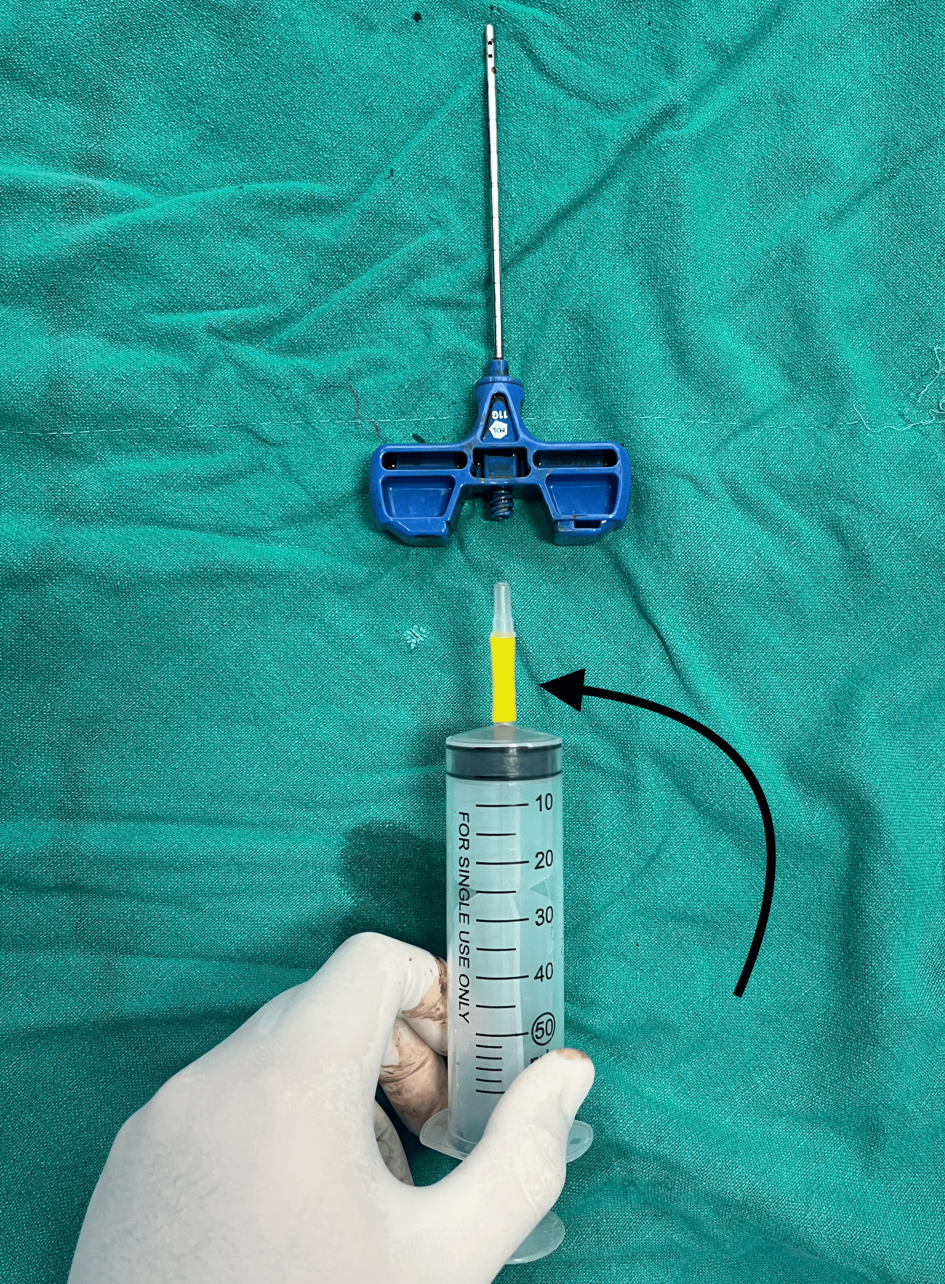
The IV drip set’s rubber bulb connector part of the fitting is over the syringe's hub (indicated by the black arrow) IV: intravenous

The rubber bulb connector part of the IV drip set is snuggly rolled over the syringe's hub, as shown in Figure 3.

**Figure 4:**
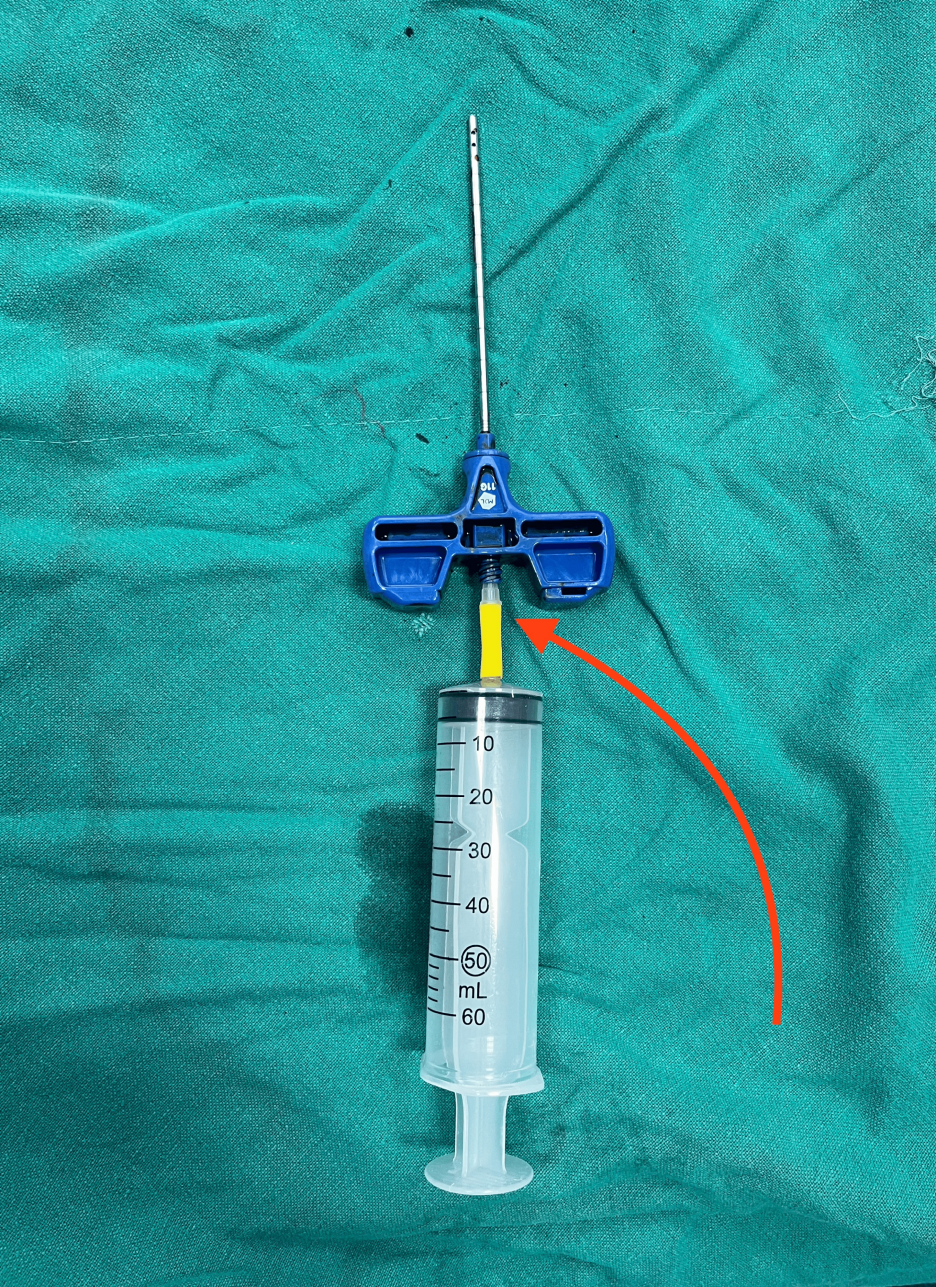
Luer connector part of the IV drip set is connected to the Jamshidi needle (indicated by the red arrow) IV: intravenous

The IV drip set's luer connector is attached to the Jamshidi needle, which is shown by the red arrow (Figure 4).

**Figure 5:**
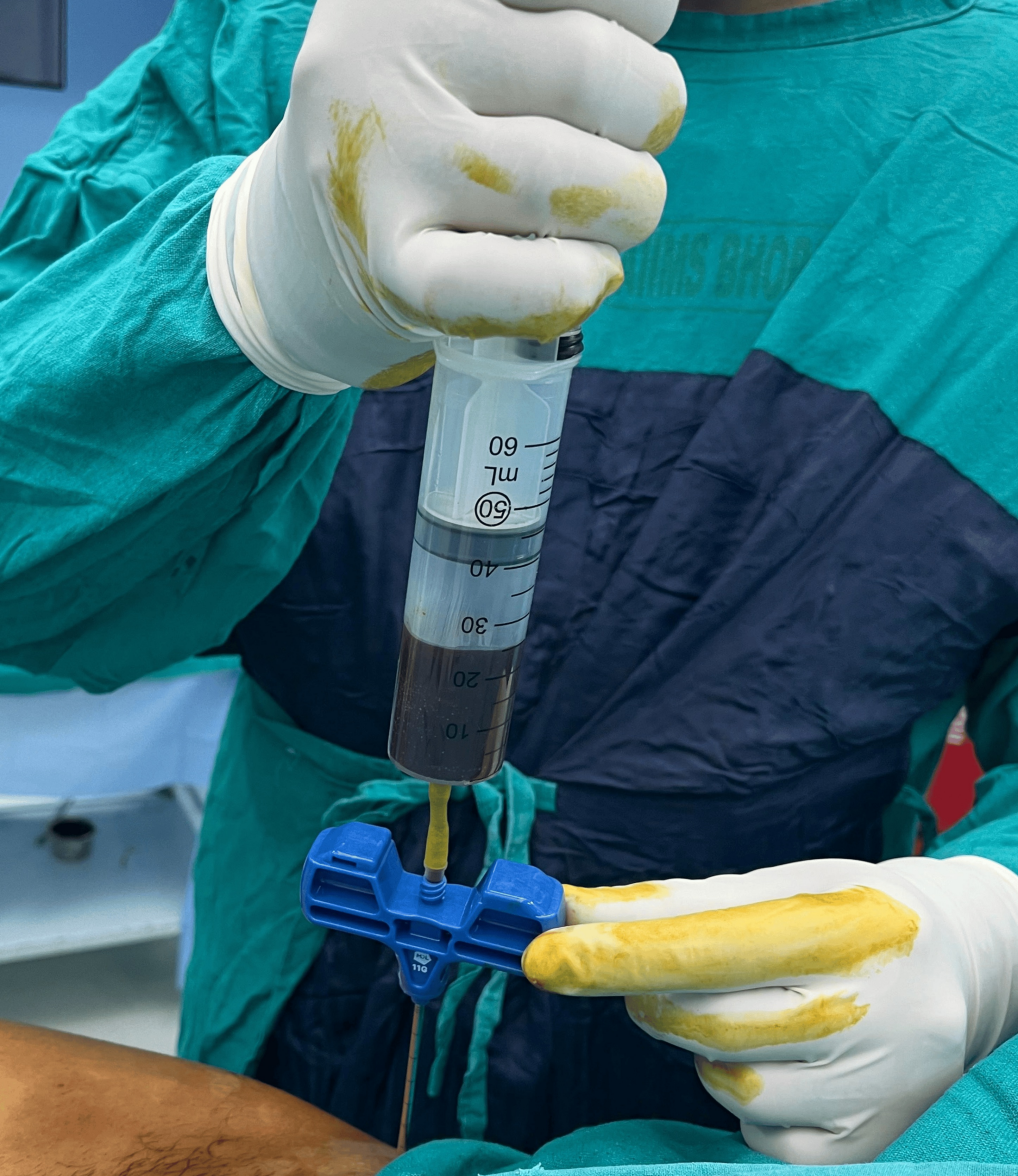
A clinical image of the modified equipment being used to collect bone marrow aspirate Donor site in this picture is right iliac crest.

Bone marrow aspirate being collected using the modified apparatus (Figure 5).

The modified apparatus successfully collected 90 ml of bone marrow aspirate in a single session. The procedure was completed without complications, and the cost of the modification was minimal. The aspirate was subsequently processed and used for BMAC application, contributing to successful bone healing.

## Discussion

The reported case highlights the utility of an innovative, low-cost modification to the bone marrow aspiration apparatus. Traditional methods for large-volume aspiration can be resource-intensive and technically challenging, particularly in settings with limited resources. The described modification addresses these challenges effectively by simplifying the apparatus assembly using easily accessible components and enhancing suction efficiency to facilitate large-volume aspiration.

The rubber bulb part serves two purposes during bone marrow aspiration. Firstly, it prevents air leakage. Secondly, since the rubber bulb part snuggly fits over the syringe, it prevents the displacement of the syringe during aspiration. The luer connector of the IV set has a tapered design and is longer than the syringe's hub. Due to this property, the luer connector of the IV set fits better over the Jamshidi needle. This design aids in creating a strong negative pressure while aspirating the bone marrow, thereby collecting more aspirate (Figure 5).

Past studies have demonstrated various methods of enhancing bone marrow aspirate quantity, like using a larger volume syringe to generate stronger negative pressure, rotating the harvesting needle after every 5-10 ml aspiration, re-angulating the needle after 30 ml aspiration, pre-flushing the syringe with anticoagulant dextrose to avoid clot formation, and using ultrasonography/fluoroscopy to localize the site of harvesting [4,5]. A study by Sugaya et al. describes the aspiration of 300 ml of bone marrow from the bilateral iliac crest. However, no specific modification/technique is described for obtaining such a large quantity of aspirate [6].

Our extensive literature research has found no published data on the modification of apparatus for enhancing the quantity of bone marrow aspirate.

The design described in this study is cost-effective and can be easily constructed using a standard IV drip set. We recommend using this design for cases requiring large quantities of bone marrow aspiration.

## Conclusions

This report underscores the potential of simple, low-cost innovations in improving medical procedures. The described modification to the bone marrow aspiration apparatus offers a practical, effective, and reproducible solution for cases requiring large-volume aspirates. Its adoption could enhance the accessibility and efficiency of therapeutic procedures like BMAC, particularly in resource-limited settings.
